# Pre-Stroke Statin Use Is Associated with Mild Neurological Deficits at the Onset of Acute Ischemic Stroke

**DOI:** 10.3390/jcdd9110396

**Published:** 2022-11-16

**Authors:** Takahisa Mori, Kazuhiro Yoshioka, Yuichi Miyazaki

**Affiliations:** Department of Stroke Treatment, Shonan Kamakura General Hospital, Kamakura City 247-8533, Kanagawa, Japan

**Keywords:** ischemic stroke, mild neurological deficits, statins

## Abstract

Pre-stroke statin use reduces infarct size. Therefore, this retrospective study aimed to investigate whether pre-stroke statin use is associated with mild neurological deficits (mND) at the onset of acute ischemic stroke (AIS). We included patients with AIS admitted to our institution within 24 h of stroke onset between 2011 and 2019. We collected data on age, sex, pre-stroke use of statins, the National Institutes of Health Stroke Scale (NIHSS) score, the serum biomarker levels, and stroke subtypes at admission. In addition, we defined mND as an NIHSS score ≤3 points. We conducted a logistic regression analysis using variables for pre-stroke statin initiation, calculated the propensity scores for pre-stroke statin use, and implemented propensity score matching (PSM). Finally, we used the McNemar test to evaluate whether pre-stroke statin administration significantly affected mND. Of 4370 patients, 2615 met our inclusion criteria. Among the 594 patients with pre-stroke statin use, 308 presented with mND. After PSM, 555 patients received pre-stroke statin treatment, while 286 patients with pre-stroke statin use presented with mND at admission (*p* = 0.0411). The binary matched pairs contingency table of mND was not symmetrical (*p* = 0.0385). Pre-stroke statin use is thus associated with mND at the onset of AIS.

## 1. Introduction

Statins (3-hydroxy-3-methylglutaryl-coenzyme A reductase inhibitors) lower low-density lipoprotein cholesterol (LDL-C) levels and the risk of cardiovascular events [1,2,3]. Additionally, they augment cerebral blood flow, exert anti-inflammatory effects [4], reduce infarct size, and improve neurological function after acute ischemic stroke (AIS) [5]. Initial neurological severity is significantly related to functional outcomes after AIS [6], and patients with mild neurological deficits (mND) at the onset of AIS are frequently discharged home [7,8,9,10]. Therefore, patients at risk of stroke may benefit from statin use [11]. Previous retrospective studies have reported equal stroke severity at admission and a better functional outcome in patients with pre-stroke statin use [12,13,14,15,16]. In atrial fibrillation-related stroke, pre-stroke statin therapy showed less neurological severity at admission and discharge [15], while post-stroke statin therapy was associated with a reduced risk of all-cause mortality [16]. Using propensity-score matching (PSM), pre-stroke statin use was found to be associated with less stroke severity and better functional outcomes [17,18,19]. Studies using PSM analysis used serum lipids levels at arrival as probable confounders to calculate the propensity scores of statin initiation [18,19]. However, statins were usually administered for primary or secondary prevention of atherosclerotic cardiovascular diseases in patients with hypercholesterolemia [20,21,22], and pre-stroke statin use influenced the serum lipid levels at the onset of AIS. Therefore, the serum lipid levels at the onset of AIS cannot be predictors of statin initiation as a response variable in patients using statins.

Statins may prevent the development of neurological symptoms at the onset of ischemic stroke. However, whether pre-stroke statin use can lead to mND at the onset of AIS remains unclear. Therefore, our retrospective cross-sectional study did not use the serum lipids levels at AIS onset as confounders for statin initiation. This study aimed to investigate whether pre-stroke statin use was associated with mND at the onset of AIS by PSM analysis.

## 2. Materials and Methods

### 2.1. Patients

We included patients with AIS admitted to our institution between April 2011 and March 2019 from the prospectively and consecutively enrolled institutional stroke registry database. Patients who were admitted to our institution 24 h after AIS onset (because information on neurological symptoms or serum biomarker levels at onset was not available), those who did not stay in the hospital to receive treatment because sufficient examinations were not performed, or those in whom pre-stroke statin use was unknown were excluded.

We retrospectively collected data from the institutional stroke registry database on age; sex; pre-stroke statin use; antiplatelets, anticoagulants, antihypertensives, and antidiabetic drugs; serum albumin, glucose, glycated hemoglobin (HbA1c), cholesterol, triglycerides, and C-reactive protein (CRP) levels; pre-stroke modified Rankin scale (mRS) and the National Institutes of Health Stroke Scale (NIHSS) scores; stroke subtypes at admission; hospitalizations; and home discharge. We defined mND as an NIHSS score ≤3 points at admission [7,8,23,24,25].

### 2.2. Statistical Analysis

Non-normally distributed continuous variables are expressed as medians and interquartile ranges. The Wilcoxon rank-sum test was used to compare unpaired groups. The Chi-square test was used to compare categorical variables. We compared variables between patients with and without pre-stroke statin use. A dummy variable was used to represent categorical data for multivariable analysis.

The propensity score was calculated for pre-stroke initiation of a statin on multivariate logistic regression analysis. We used variables that may have affected statin initiation and excluded those that might have been influenced by statin treatment, such as the serum lipid levels, stroke subtypes, NIHSS score, hospitalization, and home discharge. Patients who did and did not receive pre-stroke statins were matched one-to-one based on propensity scores. A nearest-neighbor caliper width of 0.2 multiplied by the standard deviation of the logit of propensity scores was used for matching [26,27,28,29,30]. A standardized difference of matched-patients’ variables was used to assess the adequacy of PSM [31]. We defined a standardized difference of <0.1 as an adequate variable balance between the two groups after PSM and evaluated whether pre-stroke statin use exerted a significant effect on mNS using the McNemar test [32], which evaluates symmetry on binary matched pairs contingency table of mNS. A *p*-value < 0.05 was considered statistically significant. The JMP software (version 16.2; SAS Institute, Cary, NC, USA) was used for all statistical analyses. One author (TM) had full access to all the data in the study and took responsibility for the data integrity and analysis.

## 3. Results

**Figure 1:**
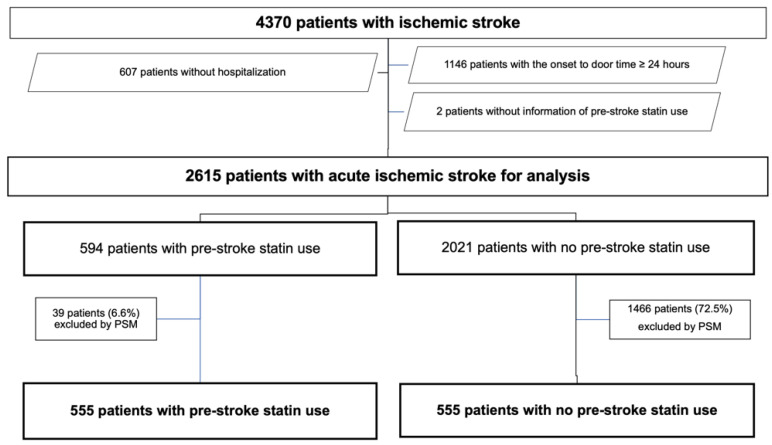
Flow chart of the patient selection process. PSM, propensity score matching.

Of the 4370 patients with AIS admitted to our stroke center during the study period, 2615 met our inclusion criteria (Figure 1). Patient characteristics at admission are summarized in Table 1.

After PSM, their median age was 79 years after PSM. Approximately 748 (67.4%) of the 1110 patients were aged ≥75 years after PSM. The median hospitalization duration was 8 days after PSM. There were differences in several variables between the unmatched pairs (Table S1). Propensity scores for pre-stroke statin initiation were calculated with logistic regression analysis (Table S2). After one-to-one PSM within a caliper of 0.169, 555 patients received pre-stroke statin treatment and 414 (74.6%) were administered strong statins. Dosage of statins followed the regulations of Ministry of Health, Labour and Welfare of Japan (Table S3). There were no significant variables between the matched pairs, and standardized differences of <0.10 showed adequate variable balance (Table 2). Their median age was 79 years after PSM. Approximately 748 (67.4%) of the 1110 patients were aged ≥75 years after PSM. The median duration of hospitalization was eight days after PSM.

There was a significant difference in the NIHSS score at admission between the matched pairs (*p* = 0.0009), and 286 patients with pre-stroke statin use presented with mND (*p* = 0.0411) (Table 3). The McNemar test showed that the binary matched pairs contingency table of mND was asymmetrical (*p* = 0.0385) (Table 4). Among the 555 patients with pre-stroke statin use, 286 presented with mND at admission and 225 (78.7%) were discharged home. In contrast, among the 555 patients without pre-stroke statin use, 252 presented with mND at admission and 159 (67.1%) were discharged home (Table S4).

After PSM, there were differences in the NIHSS score of stroke subtypes, and the median NIHSS score in cardioembolism was the largest (Tables S5 and S6). Large artery atherosclerosis had the highest frequency, while small-vessel occlusion had the lowest frequency in patients with pre-stroke statin use. In patients with LAA, the NIHSS score was lower in patients with pre-stroke statin than in those without (Table S7). In addition, the total cholesterol and LDL-C levels were lower in patients with pre-stroke statin use than in those without. However, there were no differences in the levels of high-density lipoprotein or triglycerides between the matched pairs (Table 3).

## 4. Discussion

Our results demonstrated that pre-stroke statin use was significantly associated with mND having an NIHSS score ≤3 points at AIS onset, and 78.7% of the patients with pre-stroke statin use and mND at admission were discharged home within a median duration of 8 days after admission.

We strictly defined mND as an NIHSS score ≤ 3 points, as previously reported [7,8]; however, some studies have defined mND as an NIHSS score ≤4 points [23] or ≤5 points [13,17,24]. Pretreatment with statins is associated with lower stroke severity at high (40 mg of rosuvastatin or 80 mg of any other statin) and low-to-moderate (<40 mg of rosuvastatin or <80 mg of any other statin) doses [17]. Despite administering very low doses (≤10 mg/day) of statins to our patients (Table S3) compared to other studies [15,33,34,35], pre-stroke statin use was associated with mND at AIS onset. Furthermore, the median age (79 years) observed after PSM in our patients was older than that reported in previous studies (71 [36], 66.8 [18], 70.8 [19], and 68 years [34]). In addition, our results showed that pre-stroke statin use was associated with mND and early home discharge even in aged patients (≥75 years, 67.4%).

In our patients with and without pre-stroke statin after PSM, the median NIHSS scores were 3 and 4 points (Table 3), and they were almost the same as those in the reports of the Japan Stroke Data Bank [37]. However, the median hospitalization duration of 8 days was very short compared to the median hospitalization days of 16 in men and 18 in women reported by the Japan Stroke Data Bank [37].

Previous studies have included the total cholesterol and LDL-C levels as confounders for propensity-matched analysis [18,19]. Therefore, there were no differences in the total cholesterol and LDL-C levels between the matched pairs [18]. In contrast, in our study, the total cholesterol and LDL-C levels were significantly lower in patients with pre-stroke statin use than in those without after PSM. According to the Japan Atherosclerotic Society Guidelines, LDL-C ≥ 3.62 mmol/L (140 mg/dL) is regarded as hyper-LDL cholesterolemia [18]. If statins and placebo were prospectively initiated in patients with the LDL-C ≥ 3.62 mmol/L, statins would lower the serum levels of LDL-C, but the placebo would not, and the serum LDL-C level in the statin group would probably be lower than that in the non-statin group. Therefore, our study design may be close to a prospective study design.

Statins exert pleiotropic potential neuroprotective effects, and several researchers have postulated the benefits of statin initiation in AIS [16,38]. In a previous study, statin-treated patients had significantly higher angiographic collateral scores before stroke onset compared with non-statin users [36]. Furthermore, statins appear to reduce the risk of recurrent ischemic strokes in patients with a previous stroke [39]. However, early statin therapy did not show any superiority in terms of better functional outcomes than delayed statin therapy in patients with AIS [40].

If statin treatment is associated with mND having an NIHSS score ≤3 points at AIS onset in patients with hypercholesterolemia, particularly the elderly, statins may be actively initiated. Furthermore, statin treatment may reduce neurological symptoms at AIS onset even in patients without hypercholesterolemia, as patients at risk of stroke may benefit from statin treatment regardless of their serum cholesterol levels [11].

### Limitations

Our study has several limitations. First, the study has a retrospective cross-sectional design, and selection bias might have occurred. The serum lipid levels before statin initiation were unknown and were not used to determine statin initiation. Furthermore, our study population is not representative of all patients with acute stroke. Most patients were Japanese and, therefore, the generalizability of the study outcomes to non-Japanese populations is uncertain; racial differences may exist in the efficacy of statins. Second, misclassification of the history of drugs might have introduced information bias. Third, there may be some unknown confounders for statin initiation. Therefore, a prospective and randomized controlled study is warranted to establish the effect of pre-stroke statin use on mND having an NIHSS score ≤ 3 points at AIS onset in patients with or without hypercholesterolemia.

## 5. Conclusions

Our study revealed that pre-stroke statin use is associated with mND having an NIHSS score ≤ 3 points at AIS onset. A prospective study is warranted to establish the effect of pre-stroke statin use on mND at AIS onset.

## Figures and Tables

**Table 1:** Patients’ characteristics before and after propensity score matching.

	Before PSM	After PSM
Variables	n = 2615	n = 1110
Age, years	78 (70–85)	79 (72–85)
Age ≥ 75 years, n (%)	1630 (62.3%)	748 (67.4%)
Male sex, n (%)	1463 (55.9%)	633 (57.0%)
Pre-stroke mRS	0 (0–2)	0 (0–3)
Pre-stroke statin use, n (%)	594 (22.7%)	555 (50.0%)
Hx of anti-HT drugs, n (%)	1441 (55.1%)	818 (73.7%)
Hx of diabetes drugs, n (%)	411 (15.7%)	295 (26.6%)
Hx of antiplatelets drugs, n (%)	666 (25.4%)	470 (42.3%)
Hx of anticoagulants drugs, n (%)	322 (12.3%)	166 (15.0%)
Hx of hp-EPA drugs, n (%)	72 (2.8%)	48 (4.3%)
Albumin adm, g/L	40 (37–43)	40 (37–43)
Creatinine adm, μmol/L	74.3 (61.0–92.8)	76.9 (62.8–96.4)
Glucose adm, mmol/L	6.72 (5.77–8.33)	6.83 (5.82–8.62)
HbA1c adm, % (NGSP)	5.8 (5.5–6.3)	6.0 (5.6–6.6)
CRP adm, μg/L	1200 (500–3800)	1200 (500–3600)
HDL-C adm, mmol/L	1.42 (1.17–1.73)	1.41 (1.14–1.72)
TG adm, mmol/L	1.10 (0.78–1.64)	1.13 (0.81–1.65)
TCHO adm, mmol/L	5.07 (4.34–5.79)	4.81 (4.19–5.53)
LDL-C adm, mmol/L	2.93 (2.38–3.58)	2.73 (2.22–3.36)
SVO, n (%)	429 (16.4%)	159 (14.3%)
LAA, n (%)	684 (26.2%)	333 (30.0%)
Cardioembolism, n (%)	857 (32.8%)	366 (33.0%)
ODE or UE, n (%)	645 (24.7%)	252 (22.7%)
NIHSS score adm	4 (1–11)	4 (1–10)
NIHSS score adm ≤ 3, n (%)	1203 (46.0%)	538 (48.4%)
Hospitalization, days	8 (7–9)	8 (7–9)
Discharge to home, n (%)	1240 (47.4%)	542 (48.8%)

All values except the categorical data are presented as medians (interquartile ranges). adm, at admission; CRP, C-reactive protein; HbA1c, glycated hemoglobin; HDL-C, high density lipoprotein cholesterol; hp-EPA, highly purified eicosapentaenoic acid; HT, hypertension; Hx, history; LAA, large artery atherosclerosis; LDL-C, low density lipoprotein cholesterol; mRS, modified Rankin scale; n, number; NGSP, National Glycohemoglobin Standardization Program; NIHSS, National Institute of Health Stroke Scale; ODE, other determined etiology; *p*, probability; PSM, propensity score matching; SVO, small vessel occlusion; TCHO, total cholesterol; TG, triglyceride; UE, undetermined etiology.

**Table 2:** Comparison of variables between the matched pairs after propensity score matching (n = 1110).

	After PSM			
Variable	Statin Use	No Statin Use	*p*-Value	StD
	n = 555	n = 555		
Age, years	78 (72–84)	80 (71–86)	0.0774	0.0233
Male sex, n (%)	316 (56.9%)	317 (57.2%)	0.9517	0.0036
Pre-stroke mRS	0 (0–2)	0 (0–3)	0.1656	0.0074
Hx of anti-HT drugs, n (%)	408 (73.5%)	410 (73.9%)	0.8916	0.0082
Hx of diabetes drugs, n (%)	150 (27.0%)	145 (26.1%)	0.7341	0.0204
Hx of antiplatelets drugs, n (%)	234 (42.2%)	236 (42.5%)	0.9033	0.0073
Hx of anticoagulants drugs, n (%)	85 (15.3%)	81 (14.6%)	0.7364	0.0205
Hx of hp-EPA drugs, n (%)	25 (4.5%)	23 (4.1%)	0.7679	0.0177
Albumin adm, g/L	40 (37–43)	40 (37–43)	0.3403	0.0626
Creatinine adm, μmol/L	77.8 (63.6–95.5)	76.0 (61.0–97.2)	0.1113	0.0323
Glucose adm, mmol/L	6.94 (5.83–8.77)	6.77 (5.83–8.54)	0.4978	0.0269
HbA1c adm, % (NGSP)	6.0 (5.7–6.6)	5.9 (5.6–6.6)	0.0972	0.0016
CRP adm, μg/L	1100 (400–3600)	1300 (500–3700)	0.1142	0.0454

All values except the categorical data are presented as medians (interquartile ranges). adm, at admission; CRP, C-reactive protein; HbA1c, glycated hemoglobin; hp-EPA, highly purified eicosapentaenoic acid; HT, hypertension; Hx, history; n, number; mRS, modified Rankin scale; n, number; NGSP, National Glycohemoglobin Standardization Program; *p*, probability; PSM, propensity score matching; StD, standardized difference.

**Table 3:** Comparison of variables between the matched pairs after propensity matching (n = 1110).

	After PSM			
Variables	Statin Use	No Statin Use	*p*-Value	StD
	n = 555	n = 555		
HDL-C adm, mmol/L	1.41 (1.17–1.72)	1.41 (1.12–1.74)	0.8870	0.0342
TG adm, mmol/L	1.17 (0.83–1.65)	1.11 (0.79–1.64)	0.2502	0.0122
TCHO adm, mmol/L	4.63 (3.98–5.25)	5.12 (4.42–5.77)	<0.0001	0.4583
LDL-C adm, mmol/L	2.54 (2.03–3.03)	2.99 (2.44–3.57)	<0.0001	0.3668
SVO, n (%)	73 (13.2%)	86 (15.5%)	0.2651	0.0671
LAA, n (%)	175 (31.5%)	158 (28.5%)	0.2654	0.0668
Cardioembolism, n (%)	170 (30.6%)	196 (35.3%)	0.0968	0.0999
ODE or UE, n (%)	137 (24.7%)	115 (20.7%)	0.1148	0.0946
NIHSS score adm	3 (1–9)	4 (2–11)	0.0009	0.1145
NIHSS score adm ≤3, n (%)	286 (51.5%)	252 (45.4%)	0.0411	0.1227
Hospitalization, days	8 (7–9)	8 (7–9)	0.6212	0.0412
Discharge to home, n (%)	289 (52.1%)	253 (45.6%)	0.0306	0.1299

All values except the categorical data are presented as medians (interquartile ranges). adm, at admission; HDL-C, high density lipoprotein cholesterol; LAA, large artery atherosclerosis; LDL-C, low density lipoprotein cholesterol; mRS, modified Rankin scale; n, number; NGSP, National Glycohemoglobin Standardization Program; NIHSS, National Institute of Health Stroke Scale; ODE, other determined etiology; *p*, probability; PSM, propensity score matching; StD, standardized difference; SVO, small vessel occlusion; TCHO, total cholesterol; TG, triglyceride; UE, undetermined etiology.

**Table 4:** McNemar test for a binary matched-pair contingency table of mild neurological symptoms.

		No Pre-Stroke Statin		Total	*p*-Value
		NIHSS Score Adm ≤ 3	NIHSS Score Adm ≥ 4		
Pre-stroke statin use	NIHSS score adm ≤ 3	134	152	286	0.0385
	NIHSS score adm ≥ 4	118	151	269	
Total		252	303	555	

Adm, at admission; NIHSS, National Institute of Health Stroke Scale; *p*, probability.

## Data Availability

The datasets generated and/or analyzed during the current study are available from the corresponding author upon reasonable request.

## Supplementary Materials

The following supporting information can be downloaded at: https://www.mdpi.com/article/10.3390/jcdd9110396/s1. Table S1: Comparison of variables between patients with and without pre-stroke statin use before propensity score matching (n = 2615); Table S2: Logistic regression analysis for pre-stroke statin use (n = 2615); Table S3: Statins after propensity score matching (n = 555); Table S4: Relationship among pre-stroke statin use, NIHSS score, and home discharge after propensity score matching; Table S5: NIHSS score by stroke subtypes after propensity score matching; Table S6: Comparison of NIHSS score between stroke subtypes after propensity score matching; Table S7: NIHSS score in patients with and without pre-stroke statin use by stroke subtypes after propensity score matching.

## References

[1] Baigent C., Blackwell L., Emberson J., Holland L.E., Reith C., Bhala N., Peto R., Barnes R.H., Keech A., Simes J. (2010). Efficacy and safety of more intensive lowering of ldl cholesterol: A meta-analysis of data from 170 000 participants in 26 randomised trials. Lancet.

[2] Saku K., Zhang B., Noda K., PATROL Trial Investigators (2011). Randomized head-to-head comparison of pitavastatin, atorvastatin, and rosuvastatin for safety and efficacy (quantity and quality of ldl): The patrol trial. Circ. J..

[3] Ikewaki K., Ayaori M. (2011). Strong statins as the major players for dyslipidemia in high-risk patients: Are they all the same or not?. Circ. J..

[4] Taqueti V.R., Ridker P.M. (2017). Lipid-lowering and anti-inflammatory benefits of statin therapy: More than meets the plaque. Circ. Cardiovasc. Imaging.

[5] Endres M., Laufs U., Huang Z., Nakamura T., Huang P., Moskowitz M.A., Liao J.K. (1998). Stroke protection by 3-hydroxy-3-methylglutaryl (HMG)-CoA reductase inhibitors mediated by endothelial nitric oxide synthase. Proc. Natl. Acad. Sci. USA.

[6] Sato S., Toyoda K., Uehara T., Toratani N., Yokota C., Moriwaki H., Naritomi H., Minematsu K. (2008). Baseline nih stroke scale score predicting outcome in anterior and posterior circulation strokes. Neurology.

[7] Fischer U., Baumgartner A., Arnold M., Nedeltchev K., Gralla J., De Marchis G.M., Kappeler L., Mono M.L., Brekenfeld C., Schroth G. (2010). What is a minor stroke?. Stroke.

[8] Reeves M., Khoury J., Alwell K., Moomaw C., Flaherty M., Woo D., Khatri P., Adeoye O., Ferioli S., Kissela B. (2013). Distribution of National Institutes of Health stroke scale in the Cincinnati/northern Kentucky stroke study. Stroke.

[9] Dutrieux R.D., van Eijk M., van Mierlo M.L., van Heugten C.M., Visser-Meily J.M., Achterberg W.P. (2016). Discharge home after acute stroke: Differences between older and younger patients. J. Rehabil. Med..

[10] Yaghi S., Willey J.Z., Andrews H., Boehme A.K., Marshall R.S., Boden-Albala B. (2016). The itemized nihss scores are associated with discharge disposition in patients with minor stroke. Neurohospitalist.

[11] Laufs U., Gertz K., Huang P., Nickenig G., Böhm M., Dirnagl U., Endres M. (2000). Atorvastatin upregulates type III nitric oxide synthase in thrombocytes, decreases platelet activation, and protects from cerebral ischemia in normocholesterolemic mice. Stroke.

[12] Martí-Fàbregas J., Gomis M., Arboix A., Aleu A., Pagonabarraga J., Belvís R., Cocho D., Roquer J., Rodríguez A., García M.D. (2004). Favorable outcome of ischemic stroke in patients pretreated with statins. Stroke.

[13] Elkind M.S., Flint A.C., Sciacca R.R., Sacco R.L. (2005). Lipid-lowering agent use at ischemic stroke onset is associated with decreased mortality. Neurology.

[14] Sacco S., Toni D., Bignamini A.A., Zaninelli A., Gensini G.F., Carolei A., SIRIO Study Group (2011). Effect of prior medical treatments on ischemic stroke severity and outcome. Funct. Neurol..

[15] Wankowicz P., Staszewski J., Debiec A., Nowakowska-Kotas M., Szylinska A., Turon-Skrzypinska A., Rotter I. (2021). Pre-St Therapy improves in-hospital prognosis following acute ischemic stroke associated with well-controlled nonvalvular atrial fibrillation. J. Clin. Med..

[16] Eun M.Y., Jung J.M., Choi K.H., Seo W.K. (2020). Statin effects in atrial fibrillation-related stroke: A systematic review and meta-analysis. Front. Neurol..

[17] Martínez-Sánchez P., Fuentes B., Martínez-Martínez M., Ruiz-Ares G., Fernández-Travieso J., Sanz-Cuesta B.E., Cuéllar-Gamboa L., Díaz-Domínguez E., Díez-Tejedor E. (2013). Treatment with statins and ischemic stroke severity: Does the dose matter?. Neurology.

[18] Choi J.C., Lee J.S., Park T.H., Cho Y.J., Park J.M., Kang K., Lee K.B., Lee S.J., Ko Y., Lee J. (2015). Effect of pre-stroke statin use on stroke severity and early functional recovery: A retrospective cohort study. BMC Neurol..

[19] Ishikawa H., Wakisaka Y., Matsuo R., Makihara N., Hata J., Kuroda J., Ago T., Kitayama J., Nakane H., Kamouchi M. (2016). Influence of statin pretreatment on initial neurological severity and short-term functional outcome in acute ischemic stroke patients: The fukuoka stroke registry. Cerebrovasc. Dis..

[20] Kinoshita M., Yokote K., Arai H., Iida M., Ishigaki Y., Ishibashi S., Umemoto S., Egusa G., Ohmura H., Okamura T. (2018). Japan Atherosclerosis Society (JAS) guidelines for prevention of atherosclerotic cardiovascular diseases 2017. J. Atheroscler. Thromb..

[21] Arnett D.K., Blumenthal R.S., Albert M.A., Buroker A.B., Goldberger Z.D., Hahn E.J., Himmelfarb C.D., Khera A., Lloyd-Jones D., McEvoy J.W. (2019). 2019 acc/aha guideline on the primary prevention of cardiovascular disease: A report of the american college of cardiology/american heart association task force on clinical practice guidelines. J. Am. Coll. Cardiol..

[22] Grundy S.M., Stone N.J., Bailey A.L., Beam C., Birtcher K.K., Blumenthal R.S., Braun L.T., de Ferranti S., Faiella-Tommasino J., Forman D.E. (2019). 2018 aha/acc/aacvpr/aapa/abc/acpm/ada/ags/apha/aspc/nla/pcna guideline on the management of blood cholesterol: A report of the American College of Cardiology/American Heart Association task force on clinical practice guidelines. Circulation.

[23] Laurencin C., Philippeau F., Blanc-Lasserre K., Vallet A.E., Cakmak S., Mechtouff L., Cho T.H., Ritzenthaler T., Flocard E., Bischoff M. (2015). Thrombolysis for acute minor stroke: Outcome and barriers to management. Results from the resuval stroke network. Cerebrovasc. Dis..

[24] Khatri P., Conaway M.R., Johnston K.C. (2012). Acute Stroke Accurate Prediction Study (ASAP) Investigators. Ninety-day outcome rates of a prospective cohort of consecutive patients with mild ischemic stroke. Stroke.

[25] Dargazanli C., Arquizan C., Gory B., Consoli A., Labreuche J., Redjem H., Eker O., Decroix J.P., Corlobé A., Mourand I. (2017). Mechanical thrombectomy for minor and mild stroke patients harboring large vessel occlusion in the anterior circulation: A multicenter cohort study. Stroke.

[26] Rosenbaum P.R., Rubin D.B. (1983). The central role of the propensity score in observational studies for causal effects. Biometrika.

[27] Rosenbaum P.R., Rubin D.B. (1985). Constructing a control group using multivariate matched sampling methods that incorporate the propensity score. Am. Stat..

[28] Austin P.C. (2010). Statistical criteria for selecting the optimal number of untreated subjects matched to each treated subject when using many-to-one matching on the propensity score. Am. J. Epidemiol..

[29] Austin P.C. (2011). An introduction to propensity score methods for reducing the effects of confounding in observational studies. Multivar. Behav. Res.

[30] Austin P.C. (2011). Optimal caliper widths for propensity-score matching when estimating differences in means and differences in proportions in observational studies. Pharm. Stat..

[31] Yang D., Dalton J.E. (2012). A Unified Approach to Measuring the Effect Size between Two Groups Using SAS. https://www.semanticscholar.org/paper/A-unified-approach-to-measuring-the-effect-size-two-Yang-Dalton/6cf4bd36ca4c90006a5d6563f646a391c255581b.

[32] Fagerland M.W., Lydersen S., Laake P. (2013). The McNemar test for binary matched-pairs data: Mid-p and asymptotic are better than exact conditional. BMC Med. Res. Methodol..

[33] Chou R., Dana T., Blazina I., Daeges M., Bougatsos C., Grusing S., Jeanne T.L. (2016). Statin Use for the Prevention of Cardiovascular Disease in Adults: A Systematic Review for the U.S. Preventive Services Task Force.

[34] Taguchi I., Iimuro S., Iwata H., Takashima H., Abe M., Amiya E., Ogawa T., Ozaki Y., Sakuma I., Nakagawa Y. (2018). High-dose versus low-dose pitavastatin in Japanese patients with stable coronary artery disease (real-cad): A randomized superiority trial. Circulation.

[35] Teramoto T. (2018). Extending the “lower is better” principle to Japanese and possibly other Asian populations. Circulation.

[36] Ovbiagele B., Saver J.L., Starkman S., Kim D., Ali L.K., Jahan R., Duckwiler G.R., Viñuela F., Pineda S., Liebeskind D.S. (2007). Statin enhancement of collateralization in acute stroke. Neurology.

[37] Toyoda K., Yoshimura S., Nakai M., Koga M., Sasahara Y., Sonoda K., Kamiyama K., Yazawa Y., Kawada S., Sasaki M. (2022). Twenty-Year Change in Severity and Outcome of Ischemic and Hemorrhagic Strokes. JAMA Neurol..

[38] Hong K.S., Lee J.S. (2015). Statins in acute ischemic stroke: A systematic review. J. Stroke.

[39] Tramacere I., Boncoraglio G.B., Banzi R., Del Giovane C., Kwag K.H., Squizzato A., Moja L. (2019). Comparison of statins for secondary prevention in patients with ischemic stroke or transient ischemic attack: A systematic review and network meta-analysis. BMC Med..

[40] Yoshimura S., Uchida K., Daimon T., Takashima R., Kimura K., Morimoto T., ASSORT Trial Investigator (2017). Randomized controlled trial of early versus delayed statin therapy in patients with acute ischemic stroke: Assort trial (administration of statin on acute ischemic stroke patient). Stroke.

