# Supplementary material for: Pre-Stroke Statin Use Is Associated with Mild Neurological Deficits at the Onset of Acute Ischemic Stroke
Source: J Cardiovasc Dev Dis. 2022 Nov 16;9(11):396. doi: 10.3390/jcdd9110396 (PMC9692373; doi:10.3390/jcdd9110396)
Supplement: Supplementary file 1 [file jcdd-09-00396-s001.zip › jcdd-1975695-supplementary.pdf]

Supplementary Information:

Table S1. Comparison of variables between patients with and without pre-stroke statin use before propensity score matching (n=2615)

Variables	Before PSM<br>Statin use<br>n=594	 No statin use<br>n=2021	*p*-value
Age, years	78 (72–84)	78 (69–85.5)	0.4390
Male sex, n (%)	339 (57.1%)	1124 (55.6%)	0.5298
Pre-stroke mRS	0 (0–2)	0 (0–3)	0.9052
History of anti-HT drugs, n (%)	442 (74.4%)	888 (49.4%)	<0.0001
History of diabetes drugs, n (%)	174 (29.3%)	237 (11.7%)	<0.0001
History of antiplatelets drugs, n (%)	265 (44.6%)	401 (19.8%)	<0.0001
History of anticoagulants drugs, n (%)	93 (15.7%)	229 (11.3%)	0.0059
History of hp-EPA drugs, n (%)	34 (4.80%)	13 (1.7%)	0.0008
Albumin adm, g/L	40 (37–43)	40 (38–43)	0.2398
Creatinine adm, μmol/L	77.8 (63.6–97.2)	72.5 (60.1–91.1)	<0.0001
Glucose adm, mmol/L	6.99 (5.88–8.82)	6.61 (5.77–8.16)	0.0009
HbA1c adm, % (NGSP)	6.0 (5.7–6.6)	5.7 (5.4–6.2)	<0.0001
CRP adm, μg/L	1100 (400–3400)	1200 (500–3900)	0.0328
HDL-C adm, mmol/L	1.41 (1.16–1.70)	1.43 (1.17–1.74)	0.2221
TG adm, mmol/L	1.17 (0.83–1.65)	1.07 (0.77–1.63)	0.0140
TCHO adm, mmol/L	4.60 (3.98–5.22)	5.20 (4.50–5.90)	<0.0001
LDL-C adm, mmol/L	2.53 (2.04–3.02)	3.09 (2.49–3.72)	<0.0001
SVO, n (%)	76 (12.8%)	353 (17.5%)	0.0057
LAA, n (%)	191 (32.2%)	493 (24.4%)	0.0002
Cardioembolism, n (%)	180 (30.3%)	677 (33.5%)	0.1428
ODE or UE, n (%)	147 (24.8%)	498 (24.6%)	0.9579
NIHSS score adm	3 (1–9)	2 (2–11)	<0.0001
NIHSS score adm ≤3 points, n (%)	308 (51.9%)	895 (44.3%)	0.0012
Hospitalization, days	8 (7–9)	8 (7–9)	0.1345
Discharge to home, n (%)	310 (52.2%)	930 (46.0%)	0.0081

All values, except the categorical data, are presented as medians (interquartile ranges).
adm, at admission; CRP, C-reactive protein; Glu, glucose; HbA1c, glycated hemoglobin; HDL-C, high density lipoprotein cholesterol; hp-EPA, highly purified eicosapentaenoic acid; HT, hypertension; IQR, interquartile range; med, median; LAA, large artery atherosclerosis; LDL-C, low density lipoprotein cholesterol; med, median; n, number; mRS, modified Rankin scale; n, number; NGSP, National Glycohemoglobin Standardization Program; NIHSS, National Institute of Health Stroke Scale; ODE, other determined etiology; *p*, probability; PM, propensity matching; PSM, propensity score matching; SVO, small vessel occlusion; TCHO, total cholesterol; TG, triglyceride; UE, undetermined etiology

Table S2. Logistic regression analysis for pre-stroke statin use (n=2615)

Variables	Odds ratio (95% CI)	*p*-value	AUC	BIC
		<0.0001	0.724	2529
History of antiplatelets drugs (yes/no)	2.53 (2.04–3.13)	<0.0001		
History of anti-HT drugs (yes/no)	2.20 (1.76–2.76)	<0.0001		
History of diabetes drugs (yes/no)	2.15 (1.63–2.84)	<0.0001		
HbA1c adm	1.21 (1.06–1.38)	0.0011		
History of anticoagulants drugs (yes/no)	1.50 (1.13–1.99)	0.0052		
Albumin adm	1.03 (1.01–1.06)	0.0118		
Glucose adm	0.96 (0.91–1.00)	0.0761		
CRP adm	0.99 (0.99–1.00)	0.1418		
History of hp-EPA drugs (yes/no)	1.37 (0.82–2.28)	0.2301		
Age	1.00 (0.99–1.01)	0.4465		
Creatinine adm	1.00 (0.99–1.00)	0.5655		
Male sex (yes/no)	0.95 (0.77–1.17)	0.5965		

AUC, area under the curve; BIC, Bayesian information criterion; adm, at admission; CI, confidence interval; CRP, C-reactive protein; HbA1c, glycated hemoglobin; hp-EPA, highly purified eicosapentaenoic acid; HT, hypertension; n, number; *p*, probability

Table S3. Statins after propensity score matching (n=555)

Statins	Dose	Feature	n	%
Rosuvastatin	2.5 mg/day	Strong	175	31.5
Atorvastatin	10 mg/day	Strong	155	27.9
Pravastatin	10 mg/day	Standard	117	21.1
Pitavastatin	2 mg/day	Strong	84	15.1
Fluvastatin	20 mg/day	Standard	12	2.2
Simvastatin	5 mg/day	Standard	12	2.2

n, number

Table S4. Relationship among pre-stroke statin use, NIHSS score, and home discharge after propensity score matching

	NIHSS adm	n	Discharge home	No home discharge	*p*-value
Statin use	NIHSS ≤3	286	225 (78.7%)	61 (21.3%)	<0.0001
	NIHSS ≥4	269	64 (23.8%)	205 (76.2%)	
		555	289 (52.1%)	266 (47.9%)	
No statin use	NIHSS ≤3	252	169 (67.1%)	83 (32.9%)	<0.0001
	NIHSS ≥4	303	84 (27.7%)	219 (72.3%)	
		555	253 (45.6%)	302 (54.4%)	

Adm, at admission; n, number; NIHSS, National Institute of Health Stroke Scale; *p*, probability

Table S5. NIHSS score by stroke subtypes after propensity score matching

Variables	NIHSS score adm	*p*-value
		<0.0001
SVO, n=159	3 (1–5)	
LAA, n=333	3 (1–7)	
Cardioembolism, n=366	9 (3–19)	
ODE or UE, n=252	2 (0–5)	

All values, except the categorical data, are presented as medians (interquartile ranges).
adm, at admission; LAA, large artery atherosclerosis; n, number; NIHSS, National Institute of Health Stroke Scale; ODE, other determined etiology; *p*, probability; PSM, propensity score matching; SVO, small vessel occlusion; UE, undetermined etiology

Table S6. Comparison of NIHSS score between stroke subtypes after propensity score matching

Variables	*p*-value
Cardioembolism vs. SVO	<0.0001
Cardioembolism vs. LAA	<0.0001
Cardioembolism vs. ODE or UE	<0.0001
LAA vs. SVO	0.0078
LAA vs. ODE or UE	0.0001
SVO vs. ODE or UE	0.2192

adm, at admission; LAA, large artery atherosclerosis; n, number; NIHSS, National Institute of Health Stroke Scale; ODE, other determined etiology; *p*, probability; SVO, small vessel occlusion; UE, undetermined etiology; vs, versus

Table S7. NIHSS score in patients with and without pre-stroke statin use by stroke subtypes after propensity score matching

	NIHSS score adm after PSM		
Variables	Statin use	No statin use	*p*-value
SVO, n=159	3 (1–4)	3 (1–5)	0.5425
LAA, n=333	3 (1–6)	3.5 (2–10)	0.0089
Cardioembolism, n=366	8 (2–20)	10 (3–19)	0.5044
ODE or UE, n=252	2 (0–5)	3 (1–5)	0.0770

All values, except the categorical data, are presented as medians (interquartile ranges).
adm, at admission; LAA, large artery atherosclerosis; n, number; NIHSS, National Institute of Health Stroke Scale; ODE, other determined etiology; *p*, probability; PSM, propensity score matching; SVO, small vessel occlusion; UE, undetermined etiology
